# Similarity of Data from Bee Bread with the Same Taxa Collected in India and Romania

**DOI:** 10.3390/molecules23102491

**Published:** 2018-09-28

**Authors:** Adriana Cristina Urcan, Adriana Dalila Criste, Daniel Severus Dezmirean, Rodica Mărgăoan, André Caeiro, Maria Graça Campos

**Affiliations:** 1 Department of Apiculture and Sericulture, University of Agricultural Sciences and Veterinary Medicine, 3-5 Manastur Street, Cluj-Napoca 400372, Romania; adriana.urcan@usamvcluj.ro (A.C.U.); ddezmirean@usamvcluj.ro (D.S.D.); 2 Department of Microbiology and Immunology, University of Agricultural Sciences and Veterinary Medicine, 3-5 Manastur Street, Cluj-Napoca 400372, Romania; adriana.criste@usamvcluj.ro; 3 Department of Horticulture, University of Agricultural Sciences and Veterinary Medicine, 3-5 Manastur Street, Cluj-Napoca 400372, Romania; rodica.margaoan@usamvcluj.ro; 4 Department of Life Sciences, University of Coimbra, Calçada Martim de Freitas, 3000-456 Coimbra, Portugal; andrecaeiro91@gmail.com; 5 Coimbra Chemistry Centre (CQC, FCT Unit 313) (FCTUC), University of Coimbra, Rua Larga, 3000-548 Coimbra, Portugal; 6 Observatory of Herb-Drug Interactions/Faculty of Pharmacy, University of Coimbra, Heath Sciences Campus, Azinhaga de Santa Comba, 3000-548 Coimbra, Portugal

**Keywords:** bee bread, sugar, phenolic, flavonoids, fermentation

## Abstract

Bee Bread samples from Romania and India were analysed by microscopy and High Performance Liquid Chromatography with Diode Array Detection (HPLC/DAD) and compared with pollen from the correspondent taxa. The quantification of sugars, fructose/glucose ratio, total phenolics and flavonoids was also carried out. From the results was possible to identify *Brassica* and *Eucalyptus* samples that present similar HPLC/DAD profiles with the respective ultraviolet (UV) identification of the main compounds as Kaempferol-3-*O*-glycosides and Hydrocinnamic acid derivatives. The Fructose/Glucose (F/G) ratio and the total amounts of phenolics and flavonoids was in line with the prevalence of the specie identified. These coincident fingerprints gave the identification of the samples, as was previously proposed for bee pollens. This paper relates for the first time the achievement on the taxon carried out previously only for bee pollens. It was reported for the first time that this phenolic profile remains unchanged in the case of floral pollen (hand collected), bee pollen and bee bread. Despite the biochemical transformation that occurs during the fermentation of bee bread, it seems that these phenolic compounds are not affected and remain unchanged. Also, variables such as soil and climate do not seem to influence these compounds for the kind of samples under study.

## 1. Introduction

Bee Bread (BB) is a fermented bee product made from plant pollen, honey and bee saliva. Worker bees use this product as source of protein for larvae, and for young bees. Plant pollen, collected by bees, is mixed with saliva and a small quantity of honey and stored into the cells of the honeycomb where it undergoes biochemical change. [1,2,3,4]. BB suffers different biochemical processes because of the action of moisture, temperature (35–36 °C), different enzymes from bee glandular secretions and microorganisms, allowing the transformation and preservation of the stored pollen. The opercules are filled with honey to complete the process [5].

Previous investigations have shown that BB has a better bioavailability that bee pollen because the outer layer of pollen, the exine, which is made of sporopollenin, provides resistance to the full delivery of nutrients. The exine will be responsible for the limited absorption capacity of nutrients and bioactive substances from pollen grain [6], but in BB the out layer of the pollen have been partly destroyed by fermentation and the functionally and energetically rich content of pollen can be assimilated and used more easily [7]. Different in vitro simulations of human digestion suggest that the outer layer of the pollen is only partially digested, to values that have been achieved of between 48% and 59% [8,9], which has been used by researchers as evidence to prove the resistance of the exine, even to gastric acid [10]. 

Despite these findings, another study about the digestibility of bee pollen and BB [10] have shown differences between the values for BB (79.1 g protein digested/100 g total protein) and bee pollen (63.9 g protein digested/100 g total protein). Nevertheless, being an in vitro assay, this highlights the variation of the bee pollen, and the higher bioavailability of nutrition from BB.

On the other hand, some authors claim the fact that bee pollen is a form of preservation and not nutrient conversion due to the high concentration of sugars (honey and nectar) in corbicular and hive-stored pollen (40–50%), and it was suggested that BB has evolved to be a conservation environment. The pollen stored in the comb is very acidic (pH 4) and contains 40–50% simple sugars [11,12]. One consistent change identified in BB is a decrease of starch, a substance rapidly converted by amylase secreted by the hypopharyngeal glands of the bees. Speculation that BB is a product of nutrient conversion has been constantly reinforced by the scientific community but has never been truly demonstrated [11]. 

Like bee pollen, BB is nutritionally well balanced. Its high nutritional value is given by the presence of significant quantities of proteins, lipids, microelements, vitamins, phenolic and polyphenolic compounds like natural antioxidants. The composition of BB differs slightly from that of pollen [5]. The potential application of BB as a food supplement and as a nutraceutical greatly depends on its biochemical richness, which varies upon the flora diversity of the region and the of the pollen collection [13,14]. 

The information available for BB is still rare, unlike that for bee pollen, where the scientific information on chemical composition, bioactive substances or medical and pharmaceutical applications is significant. Even most of the benefits linked with consumption of BB are based on empirical information. The biologically active substances present in BB are associated with several medicinal benefits: for example, the immune system improvement and highly active antioxidant power. BB is very useful in cases of depression, failing memory and attention, and stress. Low cholesterol levels and high quantities of calcium and magnesium recommend it in the cases of heart diseases and it helps maintain high energy levels. BB helps to regulate lipid metabolism and has also a positive effect on cardiovascular diseases [5,15,16]. BB has shown in vitro antibacterial [15,17], antioxidant [5,10,16], antitumoral [2,14] proprieties.

BB proved to be a hive product with high nutritional potentialities for consumption as a food supplement due to its rich content in proteins, essential amino acids, fatty acids, simple sugars, mineral salts, tocopherols and vitamins. BB also contains, biotin, niacin, thiamine, folic acid, phytosterols, carotenoid pigments, enzymes and coenzymes [18]. Its high bioactive properties are indicated by the presence of significant quantities of flavonoids and phenolic acids as a natural source of antioxidants. The most common flavonoids present in bee pollen by far are the flavones and flavonols which occur almost exclusively as glycosides [5,16,19,20,21].

Until now, the quantity of information available in the specialty literature about BB remains limited, with only few reports about the phenolic composition of this fermented mixture. In this work, a methodology previously developed for bee pollen identification was applied to BB samples, with the final objective to identify the main flavonoids and phenolic acids, in order to provide more data on this crude material.

This information is scarce for BB as its antioxidant properties have not been studied as much as the chemical composition of other bee products. In general, there is a consensus that BB and pollen collected by bees has a similar composition and nutritional value.

## 2. Results and Discussion

### 2.1. Microscopy Results

The Table 1 shows palynological content of bee bread (BB) samples.

### 2.2. Sugars Content

The fructose/glucose ratio for the analyzed samples was 1.46 for BB Romania—July 2016, 2.18 for BB Romania—June 2016, and 1.92 for BB Romania—April 2016. For the Indian BB the value was 1.48 and 1.29 (BB India—June 2016). However, for the samples where *Brassica* was predominant 97% (Romania—July 2016 and India—June 2016) the ratio was similar 1.46 and 1.48, respectively. The results obtained are comparable to those existing in the literature about Brassicaceae family pollen, [22,23], with exception of sucrose which was absent in both analyzed samples. For the *Eucalyptus* sp. (India—June 2016) sample a different ratio was found 1.29. Further studies will be needed to find out if this could be included as a parameter of biomarkers associated with the floral origin (hypothesis: different taxa give different ratio F/G). Fructose content in all BB samples analyzed was higher than that of glucose. Concerning minority sugars as turanose and maltose only smaller quantities were found (Table 2). 

There are only few publications about the carbohydrate profile of pollen and even less about the BB carbohydrate profile. The study of Szczesna [23] it is one of the few from the literature that identified and quantified the free carbohydrates individually and not as a difference after determinations of proteins and lipids. Szczesna [23] investigated the free sugar content of bee pollen collected in different countries (China, Poland and South Korea) using the HPLC technique. The study showed that the free sugar content of bee pollen averages 40% (dry matter). Fructose was the sugar identified in the highest amounts. It represented 46% of the total sugar content of the tested samples. Glucose was 37% of the total sugar content. The total monosaccharides (fructose and glucose) accounted about 83% of the carbohydrate fraction of bee pollen. Sucrose accounted for 8%, maltose for 7%, and trehalose and turanose for about 1% each. Samples of bee pollen from Poland were split into three groups. One of them was represented by samples with the predominance of pollen from the Brassicaceae family (over 65%), the second one corresponding to samples with the predominance of *Artemisia* pollen (over 64%) and the third group with multifloral pollen (*Rumex*, Coryphyllaceae, *Ranunculus*, *Centaurea cyanus*, *Majorana* type, *Rubus* type, *Fragaria*, *Trifolium* type, *Syringa*, *Cornus*, *Robinia* and *Salix*). No significant differences were found regarding the content of individual sugars among the sample groups under comparison. The analyzed samples with the predominant pollen of the Brassicaceae family had only a slightly lower fructose to glucose ratio. Fructose content had an average of 16.96%, glucose 12.94% (F/G ratio 1.31) and sucrose 2.94%. 

Samples of honeybee pollen from Spain, Israel, China and Romania were analysed [24] in order to quantify the free carbohydrate content. The authors reported an average of fructose content of 15.9% (Israel), 17.5% (China), 16% (Romania), 19.6% (Spain) and an average of 8.2% (Israel), 13.1% (China), 11% (Romania), 12.2% (Spain) for glucose content.

Bonvehı and Escola [22] determined the fructose/glucose ratio for the analysed bee pollen samples and had values between 1.13–1.53 for Spanish bee pollen. While [24] have obtained values of the same report between 1.53–1.66 for Spanish bee pollen, 1.94 for bee pollen from Israel, 1.33 for bee pollen from China and 1.45 for Romanian bee pollen.

### 2.3. Total Phenolic Content

**Figure 1:**
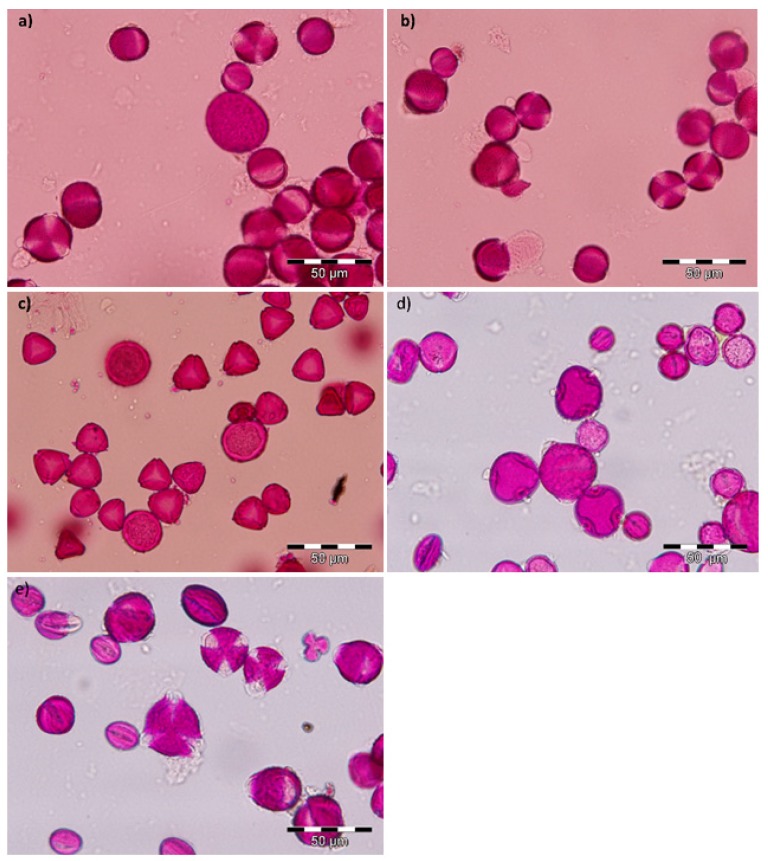
Microscopy photos of samples from BB Romania and India—showing the main pollens found. (**a**) *Brassica* sp. (Romania, July 2016), (**b**) *Brassica* sp. *(*India, June 2016), (**c**) *Eucalyptus* sp. (India, June 2016), (**d**) Fabaceae—*Trifolium*, *Tilia* sp., Fabaceae, Rosaceae, Plantaginaceae—*Plantago*, Fabaceae—*Lotus*, Asteraceae—*Taraxacum officinale*, Asteraceae, Lamiaceae, Asteraceae—*Centaurea montana* (Romania, June 2016), (**e**) *Salix* sp., *Prunus* sp., *Quercus* sp., (Romania, April 2016).

Polyphenols represent a significant group of substances with various bioactive properties including antioxidative properties. The total phenolic content in BB delimits this potential in many fresh products as in this one. The free radical scavenging activity could be used to determine the validity of bee pollen and it was specie-dependent [25]. Nevertheless, phenols and polyphenols are not the only chemicals responsible for this activity; in the present work the content of them was quantified using the Folin–Ciocaltau method and by HPLC/DAD. The amount of phenolic compound in ethanol extracts of BB obtained from the north of Romania was 6.30 ± 0.11 mg GAE/g (gallic acid equivalent/g BB sample) and in ethanol extract of Indian BB was similar, 6.41 ± 0.12 mg GAE/g (Table 3), both correspond to 97% of *Brassica* spp. (Figure 1 and Table 3). The obtained results regarding the polyphenols content were similar to the data by [10] from BB harvested in Columbia. The content of polyphenols in ethanol extracts [2] is the highest with amounts of 24.60 mg GAE/g. The content of polyphenols in all extracts obtained is in line with other researches [5,14,16,26]. The correlation with the taxon of provenience is not gave in the above-cited bibliography. 

In the sample with a high quantity of *Eucalyptus* sp. (India—June 2016) (Table 1) the content is different from *Brassica* sp. samples and this information can be used in the future for further studies in the standardization of this floral source (Table 3). 

Flavonoids are an important group of polyphenol components. In ethanol extracts, the content of flavonoids for the Romanian bee bread, in the samples, identified as *Brassica* spp. origin (97%) amounted to 29.33 ± 0.34 mg QE/g and for the similar sample from Indian BB was 30.23 ± 0.06 mg QE/g. For the other samples, the values were different (Table 3). In all extracts obtained, the content of flavonoids was in line with the results reported by Markiewicz-Zukowska et al. [2] that found a total flavonoid content between 32.72–37.15 mg Qe/g. In contrast, in the study of Zuluaga et al. [10] the total phenolic content from Columbian BB ranged from 2.5 to 13.7 mg GAE/g and total flavonoid content ranged from 1.9 to 4.5 mg Qe/g, but as pointed out before the taxa analyzed are not discussed.

It is expected that BB composition varies widely, being a fermented mixture of floral pollen collected by bees. The major variable in BB is the species composition of the pollen, which can be affected by differences in catchment area or season [20]. So far, there are only a few papers detailing the biochemical composition of BB [14,15,27,28].

In the study of Durán et al. [29] the results indicate that in all samples of BB which analysed the amount of polyphenols vary both by the type of extraction and by their geographical location. Mǎrghitaş et al. [30] also indicate that there is great variability in relation to the correspondence between antioxidant activity and total polyphenol content of pollen with different botanical origins what was previous discovery by Campos et al. [25] given the special information that contributes to the taxon identification of the matrix under analysis.

In addition, these results also coincide with those presented by Carpes et al. [31] who points out that the total amount of phenolic compounds not only varies with different extraction conditions, but also varies in different localities, which could be correlated with the floral source. Also Baltrušaityte et al. [15], indicate that the concentration of polyphenols vary according to the origin of the pollen, which clearly depends on the flora and the geographical location of it.

It was reported that the composition in phenolics [16], assessed by spectrophotometric methods, ranged between a minimum of 14 mg GAE/g BB and a maximum of 84 mg GAE/g. These values were higher than the ones reported by other authors [32] for north-east Portuguese bee pollen, and may be explained due to the higher accessibility of compounds arising from the pollen structure degradation upon fermentation of bee bread, but as we verified in this work, probably the source of pollen origin was different given a high range of possibilities. 

Regarding flavonoids, the content of flavones/flavonols in BB is much lower than the flavanones/di-hydroflavonols, ranging between 1 and 7 mg Qe/g and 10 to 89 mg Qe/g of extract, respectively. The poor flavone content was also reported by [15] in BB from Lithuania. In general, flavones and flavonols are the main flavonoids in bee pollen with the predominance of the last ones [19].

The literature data about Romanian BB total polyphenol content showed significant variability between samples from different floral species. Reported values ranged between 15.33–22.72 mg GAE/g [33]. So far there are very few studies on the chemical composition and bioactive compounds of Romanian bee bread, and no study regarding the individual content of phenolic acids.

### 2.4. Phenolic and Polyphenolic Fingerprint by HPLC/DAD

The results obtained with the samples analyzed are in line with previous results carried out in bee pollen. Spectral data for all samples were accumulated in the range 220–400 nm using DAD. The structural identifications were made according to Campos and Markham [34].

Comparing the results obtained with hand-collected pure pollen, bee pollen and BB the data remain the same. The proteins and polysaccharides present in the analyzed samples of BB did not affect the phenolic profiles obtained by HPLC/DAD analysis. With solvent programming the phenolic compounds eluted with retention times (RT) between 30 and 55 min. The first step in a tentative identification involves interpretation of the absorption spectrum to determine the compound type and compaction with reference spectra provides this information. In the range of BB examined, detection at 340 nm produced patterns comprising only flavonoids and cinnamic acid derivatives. The phenolic fingerprints of the analysed samples alone have proven to be sufficient to distinguish the pollen species represented in the present study.

**Figure 2:**
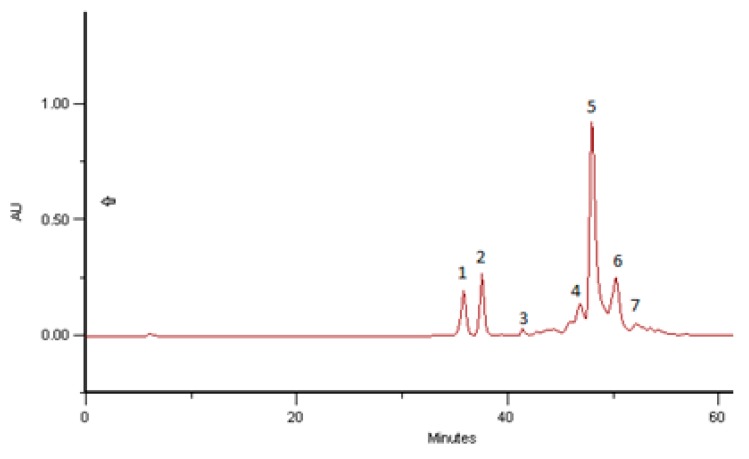

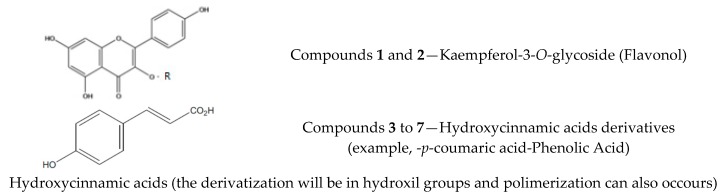
HPLC/DAD profile of BB Romanian, July 2016, corresponding to 97% of *Brassica napus* L. pollen as taxon origin. Compounds **1** and **2** are Kaempferol-3-*O-*derivatives and compounds **3**–**7** are Hydroxycinnamic acids derivatives (data in line with Campos and Markham [34]).

**Figure 3:**
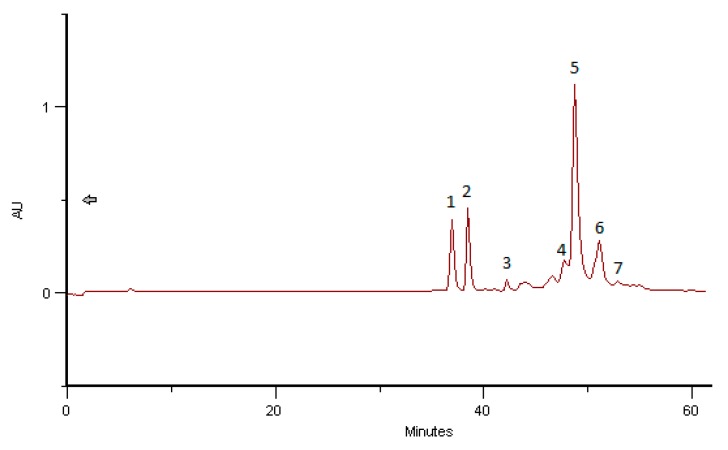
HPLC/DAD profile of BB India, June 2016 corresponding to 98% of *Brassica napus* L. (rape) pollen as taxon origin. Compounds **1** and **2** are Kaempferol-3-*O*-derivatives and compounds **3**–**7** are Hydroxycinnamic acids derivatives (data in line with Campos and Markham, 2007 [34]).

**Figure 4:**
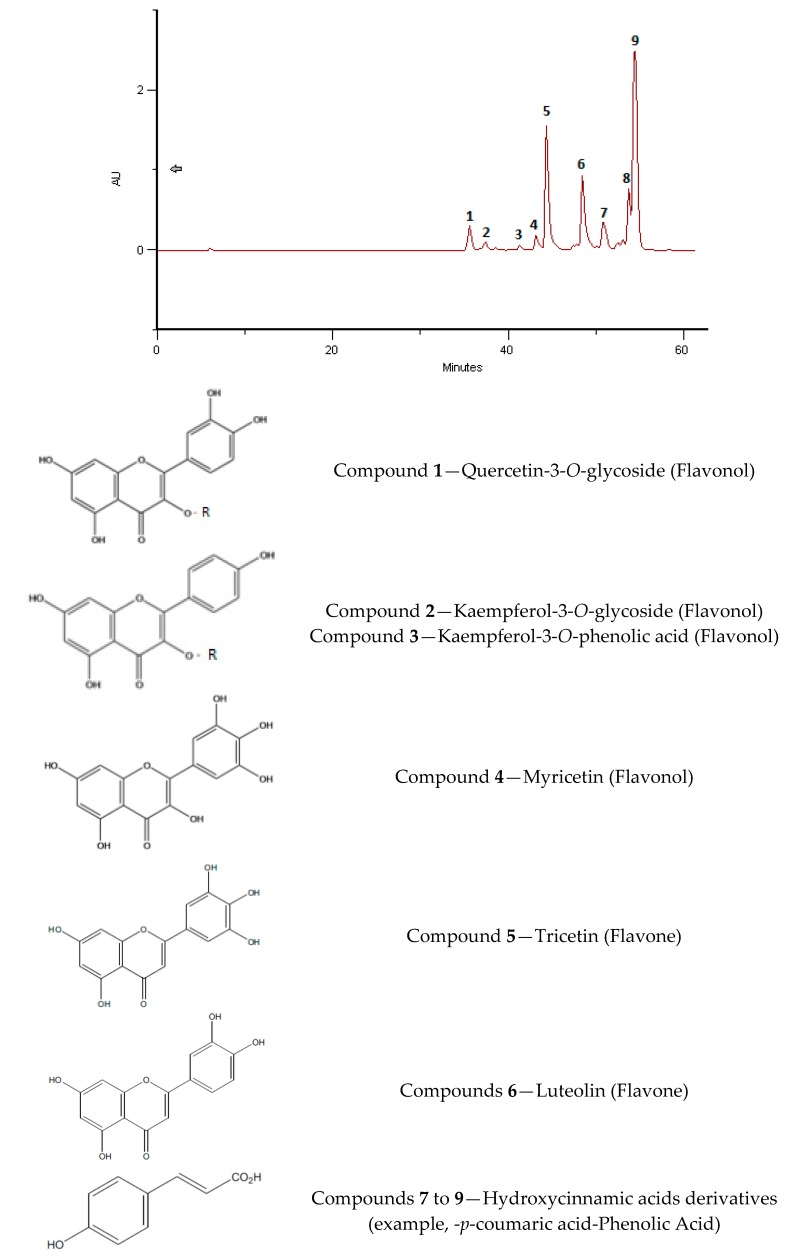
HPLC/DAD profile of BB India, June 2016 that correspond to *Eucalyptus* sp. (91%), *Citrus* sp. (7%), *Asteraceae* (2%). Compound **1** = quercetin-3-*O-*derivative, compound **2** = Kaempferol-3-*O-*derivative, compound **3** = Kaempferol-3-*O-*derivative, probably with a Hydroxycinnamic acids derivative in C3; compound **4** = Myricetin, compound **5** = Tricetin and compound **6** is Luteolin. Compounds **7**–**9** are Hydroxycinnamic acids derivatives (data in line with Campos and Markham [34]).

**Figure 5:**
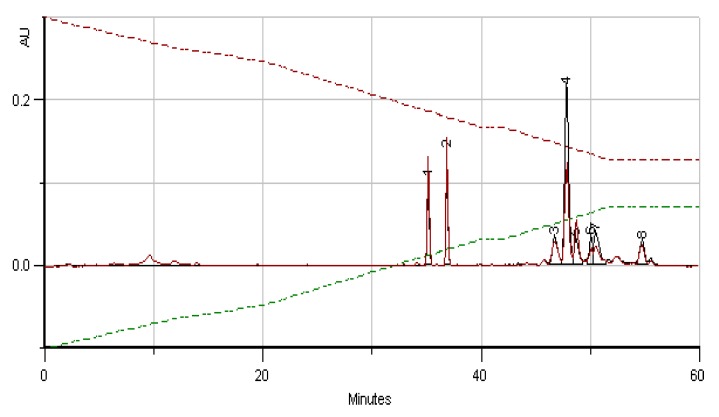
HPLC/DAD profile of pure rape pollen sample (these analyses were carried out with pollen from Slovakia). The profile is identical and the ultraviolet (UV) spectra of each compound have the similar correspondence as presented in Figure 2 and Figure 3.

**Figure 6:**
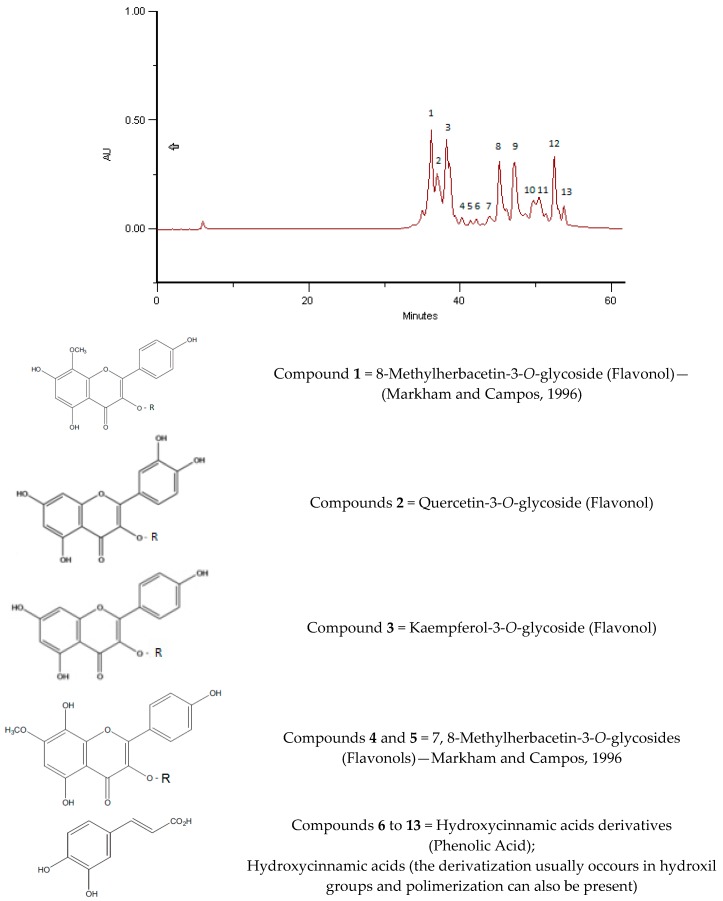
HPLC/DAD profile of BB Romania—April 2016 that correspond to Salicaceae—*Salix* sp. (61%), Rosaceae—*Prunus* sp. (26%), Fagaceae—*Quercus* sp. (13%). Compound **1** = 8-methylherbacetin-3-*O*-derivative (Markham and Campos, 1996), compound **2** = quercetin-3-*O*-derivative, compound **3** = Kaempferol-3-*O*-derivative, compounds **4** and **5** = 7, 8-methylherbacetin-3-*O*-derivative (Markham and Campos [37]). All additional compounds are phenolic acid derivatives (data in line with Campos and Markham [34]).

**Figure 7:**
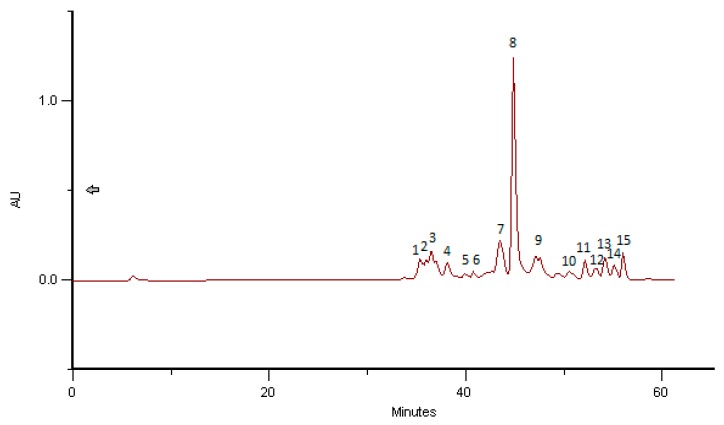
HPLC/DAD profile of BB Romania, June 2016 that correspond to *Fabaceae*—*Trifolium* (34.6%), *Tiliaceae*—*Tilia* sp. (22%), *Fabaceae* (13%), *Rosaceae* (7.5%), *Plantaginaceae*—*Plantago* (5%), *Fabaceae*—*Lotus* (7.5%), *Asteraceae*—*Taraxacum officinale* (3%), *Asteraceae* (3%), *Lamiaceae* (2.9%), *Asteraceae*—*Centaurea montana* (1.5%). Compound **1** = kaempferol-3-*O*-derivative, compounds **2** and **3** = quercetin-3-*O*-derivative, compound **4** = isorhamnetin-3-*O*-derivative. All the remain compounds are phenolic acid derivatives (data in line with Campos and Markham [34]).

The phenolic profiles obtained were simple, composed of flavonol, flavones and hydroxycinnamic acids derivates (Figure 2, Figure 3, Figure 4, Figure 5, Figure 6 and Figure 7).

Figure 2 and Figure 3 give the HPLC/DAD profile of BB from Romanian collect in July 2016 and from India collected in June 2016, corresponding both to 97% of *Brassica* sp. pollen as the main taxon*.* The respective UV-spectra of compounds **1** and **2** correspond to C3 glycosylated flavonols, both Kaempferol-3-*O*-derivatives. All the other compounds are hydroxycinnamic acids derivatives (data in line with Campos and Markham [34]) with a high probability of polymerized structures as spermidine polyamides.

It was identified in rape bee pollen three flavonols including quercetin-3-*O*-(2″-*O*-glucopyranosyl)-glucopyranoside (in very low amounts), quercetin-3-*O*-(2″-*O*-glucopyranosyl)-rhamnopyranoside and kaempferol-3-*O*-(2″-*O*-glucopyranosyl)-glucopyranoside [35]. They also identified and *N*′,*N*″-di-*p*-coumaroylspermidine in rape bee pollen crude extracts using high performance liquid chromatography coupled to electrospray ionisation and quadrupole time-of-flight-mass spectrometry (HPLC-ESI-QTOF-MS/MS). These last compounds are polyamines including the hydroxycinnamic acid, *p*-coumaric linked in the *N* of spermidine. Under UV analysis, only the structure of the phenolic acid is responsible for the absorption and the spectra obtained. The profile is not similar to the one that we present in this paper because the methodology applied was different. Rape bee pollen suffers a previous “wall-breaking treatment” and only after was extracted with 75% ethanol under reflux at a solid/liquid. The authors also prepare the final crude residue after a purification with petroleum ether to remove liposoluble impurity and, finally a water-saturated n-butanol clean-up. Nevertheless, in the results obtained by HPLC-ESI-QTOF-MS/MS the MS spectra of the three flavonols, does not correspond with the further identification. 

In Figure 4 we show compounds **1** and **2** as quercetin and kaempferol glycosides, respectively, and as can be seen the UV spectra is different due to the hydroxylation in B-ring of flavonoid structure.

In Figure 4, the HPLC/DAD profile corresponds to a Eucalyptus pollen source with the characteristic flavonoids, in this case two flavonols quercitine-3-*O*-sophoroside (compound **1**) and myricetin (compound **4**) and the two flavones, tricetin (compound **5**) and luteolin (compound **6**). Tricetin is a biomarker for Myrtaceae [36]. The kaempferol derivatives (compounds **2** and **3**) could be from citrus pollen. Nevertheless, until now we have not had access to this kind of pollen to confirm the identification. 

As can be seen in Figure 5, there is a huge similarity of the HPLC/DAD profile of phenolic/polypfenolic compounds from pure rape pollen samples analysed years ago and from Slovakia (data not published yet) with the BB profiles of samples from Romania and India in July and June 2016, respectively. These coincident fingerprints give the identification of the samples, as was previous proposed for bee pollens by Campos et al. [19]. This paper gives for the first time some achievement to the taxon carried out previous only for bee pollens. 

In contrast to the specie-specific data show above from samples with high percentage of one specie, in Figure 6 the HPLC/DAD profile obtained with BB from Romania collected in April 2016 corresponds to a complex mixture of Salicaceae—*Salix* sp. (61%), Rosaceae—*Prunus* sp. (26%) and Fagaceae—*Quercus* sp. (13%). The UV-spectra correspond to 8-methylherbacetin-3-*O*-derivative (Compound **1**), quercetin-3-*O*-derivative (Compound **2**), Kaempferol-3-*O*-derivative (Compound **3**), compounds **4** and **5** = 7,8-methylherbacetin-3-*O*-derivative (Markham and Campos [37]). The additional compounds are phenolic acid derivatives (data in line with Campos and Markham [34]). In this case it was not possible to find a correct correspondence to a high provenance of pollen because, in fact, it does not exist. For pollen from Romania, June 2016, shown in Figure 7, the correspondence was even more difficult for some reason, as explained above.

As shown, through the results from various samples, different polyphenolic profiles were obtained with BB from Romania and India. Firstly we compared them and then compared them with pure pollen samples, hand collected (data from other essays that belong to the data base of the Pharmacognosy laboratory—University of Coimbra, Portugal, some of them already published [19,20,38]). The BB samples analyzed were almost pure in *taxon* (samples collected in Romania July 2016 and India June 2016). It contained pollen from *Brassica napus* L*.* almost entirely, 97%, Romanian bee bread, respectively 98% Indian bee bread. In order to confirm the botanical origin of the bee bread, microscopic analysis was carried out as well as the HPLC/DAD analyses of floral pollen collected directly from *Brassica* plants collected in Portugal.

BB samples that had *Brassica* majority pollen (>90%) have been analyzed as well as pollen collected directly from *Brassica* family. An attempt was made to find out by means of chromatographic comparisons of natural plant pollen, bee pollen and bee bread, if bee saliva and the fermentation of bee pollen affects the phenolic composition by enzymic action. From the data obtained, the profile was maintained in the three samples, from Romania, India and even from Slovakia. Similar HPLC/DAD profiles were obtained with bee pollen samples from Sultanate of Oman (in 2010) and from Brazil (in 2012) (data not shown).

In the tested samples of *Brassica* bee bread, five Hydroxycinnamic acid derivatives, as well as two flavonoids, Kaempferol-3-*O*-glucoside, were found. The quantitative analysis was performed based on calibration curves plotted for the reference substances: Hydroxycinnamic acids (R = 0.997) and rutin (R = 0.998). For all calibration curves, the level of significance *p* was lower than 0.001 (*p* < 0.001). The content of the compounds measured in BB extracts is presented in Table 4. 

Hydroxycinnamic acid derivatives were present in these samples in the greatest amounts while flavonoids, such as Kaempferol-3-*O-*glycosides, were present in a lower proportion. Strong antioxidative properties of hydroxycinnamic acids derivates were described by other authors [39], and for these samples the potential could be similar. This will be explored in further studies. The proportion of the two flavonoids in the samples was similar, and corroborated the identification of the same taxon*.*

It was reported for the first time that this phenolic profile remains unchanged in the case of floral pollen (hand collected), bee pollen and bee bread. Despite the biochemical transformation that occurs during the fermentation of bee bread, it seems that these phenolic compounds are not affected and remain unchanged. Also, variables such as soil and climate do not seem to influence these compounds for the kind of samples under study.

Honeybee pollen seems to accumulate a species-specific selection of flavonoids [40]. Using HPLC/DAD, Campos et al. [19] pointed out that each botanical species studied gave a unique fingerprint of polyphenolic compounds (flavonoids plus other phenolics such as hydroxycinnamic acid derivatives). Flavonoids are generally recognized as reliable chemotaxonomic markers of plants [41]. Evidence obtained by Campos et al. [19] suggests that this also applies for bee pollen flavonoids. It was found that pollen from *Ulex europaeus* plants from the Bay of Plenty (New Zealand) has the same polyphenolic profile as did the plants growing in Lower Hutt (New Zealand) 400 km away and even from plants growing in Portugal. The same situation was reported for *Eucalyptus globulus* when pollen analysis from plants grown in different geographic areas showed an identical phenolic profile. In the main pollen samples, flavonoid profiles identified from pollen extracts are characterized by the presence of flavonoid glycosides mainly from quercetin, kaempferol, isorhamnetin and/or myricetin [19].

It is known that phenolic composition of the plants species depends on its chemotype and on various environmental factors; therefore, it would be reasonable to assert that the phenolic composition of BB depends on its floral origin [15]. The authors reported that seven samples of BB, from the nine tested, contained significant amounts of *p*-coumaric acid. BB also contained higher amount of kaempferol than honey. Instead, chrysin and apigenin were present in BB in trace levels. The reported results clearly demonstrated that HPLC peak areas of the polyphenolic compounds in honey and BB extracts and, therefore, the quantity of phenolic compounds, varied in a very wide range. 

In fact, the content in phenolic compounds of BB has been very poorly studied and has been reported by few authors who have used different methods of identification and quantification [2,28,29]. Most existing literature only mentions the total phenolics content measured by the Folin–Ciocalteu colorimetric assay [10,29] and do not provide detailed characterization of individual phenolic compounds. A few studies of BB samples from Poland, Georgia and Portugal identify some individual phenolic compounds, but all of them have used different analytical approaches. The first to present data on the biochemical composition of BB was Isidorov et al. [28]. In this study, the gas chromatography with mass spectrometry (GC-MS) method was used, and six flavonoids were identified (kaempferol, chrysin, naringenin, isorhamnetin and apigenin) and four phenolic acids (4-hydroxybenzoic acid, *p*-coumaric, ferulic and caffeic acid). Markiewicz-Zukowska et al. [2] identified, using the GC-MS method, kaempferol and apigenin. The presence of the flavonoids as naringin, rutin and quercetin, was reported in Georgian BB samples, using high performance liquid chromatography with UV-VIS detector (HPLC-UV-VIS) [42]. Rutin [quercetin-3-*O*-rhamnosyl (1–6) glucoside] is often confused with quercetin-3-*O*-rhamnosyl (1–2) glucoside. In terms of chromatography is impossible to determine the configuration of the sugars linkage among them. As was published before, the main sugar linkage in flavonoid sugars found in pollen is 1–2 [37].

In BB from Portugal, up to 32 different flavonoids, using HPLC-DAD-ESI/MS were identified [14]. The phenolic compounds found in BB were mainly flavonol derivatives, such as quercetin, kaempferol, myricetin, isorhamnetin and herbacetin glycosides. They found several compounds and classified various as rutinosides, despite the situation cited above. Quercetin-3-*O*-rutinoside, kaempferol-3-*O*-rutinoside, quercetin-3-*O*-glucoside were found in all the studied samples, while myricetin-3-*O*-glucoside, isorhamnetin-3-*O*-rutinoside and isorhamnetin-3-*O*-glucoside were only detected in some BB samples. In this study, all these compounds were positively identified depending to their retention time, mass and ultraviolet-visible (UV-vis) characteristics in comparison with commercial standards. Once again, using this methodology it is impossible to classify the sugar linkage. It is correct to state that this compound had rhamnose and glucose as glycosidic moiety but is not classified as rutinose [rhamnosyl (1–6) glucoside] because in this case the 1–6 linkage is implicit.

Bees foraging preference is still being discussed; recent studies shows that honey bees prefer diets that reflect the proper ratio needed for optimal survival and homeostasis. The nutritional quality of pollen, mainly linked to the protein and amino acid content, is only in part the main influencing factor, and other non-nutritional ones are cited as involved, such as plant resource availability, color, odor and morphology of flowers [43]. In effect, honeybees do collect and consume pollen and pollen-like substances with little or no nutritive value [44]. Honeybee pollen generally shows characteristic quantities of total polyphenols due to its different botanical and geographical origin [45]. Flavonoids are minor compounds, but of great importance in bee pollen and thus bee bread. These compounds influence the aspect of the pollen grain (pigmentation) and taste (astringency and bitter taste) [46]. The most common flavonoids founded in bee pollen are the flavones and flavonols which occur almost exclusively as glycosides. Aglycones have in general simple structures such as quercetin or kaempferol, but their glycosylation patterns have structures that are more complex than those found in the vegetative plant parts [47] as discussed above.

## 3. Materials and Methods 

### 3.1. Samples Collection

BB samples were collected as follows:

In the spring of 2016 (April) and in the summer of 2016 (June and July) from *Apis mellifera* hives located in the north-west of the Transylvania area, Romania, and in the summer of 2016 (June) from *Apis dorsata* in the Bangalore area, India.

The BB was provided by the local beekeepers. To maintain BB quality, samples were stored in a freezer (−18 °C) until they were analysed.

### 3.2. Standards and Reagents

Caffeic acid (3,4-Dihydroxycinnamic acid) and rutin (quercetin-3-*O*-rutinoside) were obtained from Merck (Darmstadt, Germany). Acetonitrile was purchased from Merck (Darmstadt, Germany); Water MilliQ pH = 2.4 (with *o*-phosphoric acid from Sigma Aldrich, Darmstadt, Germany).

### 3.3. Microscopic Analysis

The floral origin of multifloral BB samples was determined by optical microscope examination, performed at 40× magnification (microscope Nikon Eclipse). Palynological analysis was based on the European standard [48] without acetolysis and adapted for BB [49]. For the identification of pollen type existent in BB samples, pollen atlas or reference slides prepared from anthers of flowers were used. Most of the BB samples were multifloral samples and to establish the occurrence percentages of each pollen type at least 1000 pollen grains were counted in different microscopy fields. Regarding the taxonomic identification, this was done at the lowest level possible, depending on the difficulty level encountered.

### 3.4. Sugar Profiles

For carbohydrate analysis was used the method developed by [50] adapted for BB. 2.5 g of each sample were dissolved in water and after that was transferred quantitatively into a 500 mL volumetric flask, containing 12.5 mL methanol and filled up to the volume with water. The solution was filtered through a 0.45 µm Millipore syringe filter, collected in vials and stored at 4 °C until further analysis. A Shimadzu Liquid Chromatograph with refractive index detector (HPLC/IR) was used. Chromatographic separation of carbohydrates was performed on Altima Amino 100 stainless steel column, length 250 mm and diameter 4.6 mm (Alltech, Nicholasville, KY, USA), particle size of 5 μm. The temperature inside the column was maintained at 30 °C by means of a CTO-10 AS thermostatic furnace. Column pressure was 6.3 MPa. The mobile phase was acetonitrile: water (75:25 *v*/*v*) at a flow rate of 1.3 mL/min and it was filtered through a membrane filter (0.45 µm) before chromatographic analysis. The injection volume was 10 µL. A calibration curve was plotted using standard solutions of different concentrations (0.5–80 mg/mL). The linear regression factor of the calibration curves was higher than 0.9982 for all the standard sugars solutions. Free sugars were quantified by comparison of the peak area obtained with those of standard sugars. The results were expressed as g/100 g honey for each sugar.

### 3.5. Total Phenolic and Flavonoids Content 

#### 3.5.1. Preparation of Extracts

The BB samples (1 g) were individually extracted for three times with 5 mL of methanol solvent at the room temperature for 1 h. After sonication (15 min), maceration the resulting mix was centrifuged (15,269 g), for 10 min. and the supernatants were collected and stored until analysis (4 °C).

#### 3.5.2. Total Phenolic Content by Folin–Ciocateau Method

The content of total phenolics was determined through the Folin–Ciocateau method [51]. Briefly, 100 µL Folin–Ciocalteu reagent, diluted 1:10 with de-ionised water (0.2 M—with respect to acid), was added to 10 µL of BB extracts and mixed with 80 µL sodium carbonate (Na_2_CO_3_) solution (1 M). After 20 min. the absorbance of the resulting blue color mixt was measured at 630 nm. For the quantification, a calibration curve of gallic acid was prepared with solutions in the range of 0.025–0.15 mg/mL (R^2^ = 0.9992). The results were expressed as mg of GAE (Gallic acid equivalent) per g of extract. The assays were run in triplicate.

#### 3.5.3. Total Flavonoid Content 

Previously the flavonoid fraction was purified, by liquid–liquid extraction, with ethyl acetate. Each sample was suspended in ethanol 50%, after the alcoholic solvent was evaporated and the water solution was extracted with the same amount of ethyl acetate (1:1) for three times. All the organic fractions were associated and evaporated under vacuum. The dry residue was recovered in methanol for the quantification. Total flavonoids were measured using an aluminum chloride colorimetric assay [30,52] adapted for the 96 well microplate reader (Synergy™ HT BioTek Instruments, Winooski, VT, USA), using quercetin as reference standard. An exact volume of 25 µL of appropriately diluted sample o was added to 100 µL distilled water and 10 µL sodium nitrate (NaNO_2_) solution 5%. After 5 min, 15 µL aluminum chloride (AlCl_3_) 10% was added. At 6 min, 50 µL sodium hydroxide 1 M (NaOH) was added to the mixture. Immediately, the reaction flask was diluted to volume with the addition of 50 µL of distilled water and thoroughly mixed. Absorbance of the mixture, pink in colour, was determined at 510 nm. For the quantification, a calibration curve of quercetin was prepared with solutions in the range of 0.025–0.2 mg/mL (R^2^ = 0.9987). The results were expressed as mg of Qe (quercetin equivalent) per g of extract. The assays were run in triplicate using a SPECTRAmax PLUS 384 (microwell) spectrophotometer. 

### 3.6. Phenolic and Polyphenolic Fingerprint by HPLC/DAD

#### Sample Extraction

To determine phenolic and polyphenolic profile in a way to assess the botanical origin of BB a high-performance liquid chromatography with a diode array detector (HPLC/DAD) was performed. The phenolic profiles of BB samples were compared with those carried out by hand collected pollen from the plants (including *Brassica* sp.) as has been previously proposed by other authors [19,53]. BB samples were extracted with ethanol:water (50% *v*/*v*) in a proportion of 10 mg of sample/mL in a 1.5 mL Eppendorf tube and sonicated for 1 h. The resulting mix was centrifuged (15,269 g), for 5 min. and the supernatants were collected, microfiltered and used for HPLC/DAD analysis [19]. Extracts were analyzed on a Gilson HPLC System, UV detector Gilson 170 and Waters Spherisorb S500D52 (5 µm) column (4.6 × 250 mm) by an acidified water–acetonitrile gradient as detailed below.

For the determination of flavonoids (among other polyphenols) and phenolic acids from BB extracts a 100 μL injection volume was used. The solvents were represented by water adjusted to pH 2.5 with orthophosphoric acid (A) and acetonitrile (B). It was used a linear gradient starting with 100% A, decreasing to 91% over the next 12 min to 87% over the next 8 min and to 67% over the next 10 min. The solvent was holding at this composition for 2 min, phase A was decreased to 57% over the next 10 min, and then held at this level until the end of the 60 min analysis. All spectral data were accumulated in the range 220–400 nm. The linearity of the detector response was checked with the standards, Hydroxycinnamic acids and quercetin-3-*O*-rutinoside (rutin) [19].

## 4. Conclusions

In the case of BB samples, comparison with previous literature data is difficult, since few data are available. The results obtained for BB samples were compared with previous studies made on *Brassica* pollen from other countries (Brazil, Portugal, Slovakia and the Sultanate of Oman) and all gave an identical HPLC/DAD profile of phenolic and polyphenolic compounds. Another important aspect is that the concentration of flavonoids and phenolic acids was almost identical in all analysed samples and even similar for the samples with the same floral source. All of the results support the statement that polyphenols are chemotaxonomic markers for plants and make HPLC/DAD analysis of the polyphenolic profile an analysis capable of identifying the botanical origin of a sample by simply analysing the spectrum obtained that gave a unique fingerprint, even with bee bread.

There are still few studies of the palynologic spectrum of bee pollen and bee bread in Romania and India and these have only been sparsely reported. From what is known so far, studies regarding the phenolic profile of this matrix from Romania and India do not exist. The present work provides important additional data about the pollen flora collected and stored by honeybees in these countries, and variables such as soil characteristics and climate do not seem to affect the phenolic profile of analysed samples. The study can be replicated with different bee bread samples from various countries, once it confirms previous findings that the botanical origin influences the polyphenol content of bee pollen and the specific occurrence of them in the monofloral pollen loads, but also the fact that polyphenols can be considered an extremely useful tool for identifying the botanical origin of pollen. We can conclude that the content of polyphenols varies between species, but not within the same species.

## Figures and Tables

**Table 1:** Palynological content of bee bread (BB) samples.

Sample	Floral Composition (Botanical Name, %)
BB Romania—July 2016	Brassicaceae—*Brassica* sp. (97%), *Poaceae* (3%)
BB India—June 2016	Brassicaceae—*Brassica* sp. (98%), 2% Unknown pollen
BB India—June 2016	Myrtaceae—*Eucalyptus* sp. (91%), Rutaceae—*Citrus* sp. (7%), Asteraceae (2%)
BB Romania—June 2016	Fabaceae—*Trifolium* (34.6%), Tiliaceae—*Tilia* sp. (22%), Fabaceae (13%), Rosaceae (7.5%), Plantaginaceae—*Plantago* (5%), Fabaceae—*Lotus* (7.5%), Asteraceae—*Taraxacum officinale* (3%), Asteraceae (3%), Lamiaceae (2.9%), Asteraceae—*Centaurea montana* (1.5%)
BB Romania—April 2016	Salicaceae—*Salix* sp. (61%), Rosaceae—*Prunus* sp. (26%), Fagaceae—*Quercus* sp. (13%)

**Table 2:** Sugar content and ratio of F/G in the various samples of BB analyzed.

Sample	Fructose (%)	Glucose (%)	Sucrose (%)	Turanose (%)	Maltose (%)	F/G
BB Romania—July 2016	19.32 ± 0.01	13.19 ± 0.01	nd	0.66 ± 0.01	0.89 ± 0.01	1.46
BB India—June 2016	18.79 ± 0.06	12.66 ± 0.04	nd	0.65 ± 0.01	0.82 ± 0.01	1.48
BB India—June 2016	19.58 ± 0.03	15.13 ± 0.02	nd	0.70 ± 0.02	1.00 ± 0.01	1.29
BB Romania—June 2016	13.97 ± 0.05	6.40 ± 0.010	nd	0.56 ± 0.02	0.82 ± 0.02	2.18
BB Romania—April 2016	19.29 ± 0.03	10.07 ± 0.02	nd	0.87 ± 0.01	0.91 ± 0.02	1.92

nd = not detected.

**Table 3:** Content in phenol and polyphenolic compounds.

Sample	Total Polyphenols (mg GAE/g)	Total Flavonoids (mg Qe/g)
BB Romania—July 2016	6.30 ± 0.11	29.33 ± 0.34
BB India—June 2016	6.41 ± 0.12	30.23± 0.06
BB India—June 2016	5.67 ± 0.09	34.46 ± 0.09
BB Romania—June 2016	7.57 ± 0.16	23.64 ± 0.1
BB Romania—April 2016	12.83 ± 0.08	36.19 ± 0.05

**Table 4:** Content of the flavonoids and Hydroxycinnamic acids derivatives identified in BB extracts by HPLC/DAD. The quantification was carried out at 260 nm.

Sample	Name of the Substance	Time of Retention	Bee Bread mg/g
BB Romania, 2016, *Brassica* sp.	Kaempferol-3-*O*-glycoside	35.83	2.68
	Kaempferol-3-*O*-glycoside	37.64	2.33
	Hydroxycinnamic acid derivative 1	42.11	0.04
	Hydroxycinnamic acid derivative 2	47.61	0.80
	Hydroxycinnamic acid derivative 3	48.69	5.59
	Hydroxycinnamic acid derivative 4	50.99	1.77
	Hydroxycinnamic acid derivative 5	54.82	0.10
BB India, 2016, *Brassica* sp.	Kaempferol-3-*O*-glycoside	35.83	2.57
	Kaempferol-3-*O*-glycoside	37.64	2.21
	Hydroxycinnamic acid derivative 1	42.19	0.03
	Hydroxycinnamic acid derivative 2	47.74	0.58
	Hydroxycinnamic acid derivative 3	48.72	5.61
	Hydroxycinnamic acid derivative 4	51.08	1.62
	Hydroxycinnamic acid derivative 5	54.94	0.08
BB India, 2016, *Eucalyptus* sp.	Quercetin-3-*O*-sophoroside	35.61	2.40
	Kaempferol-3-*O*-glycoside	37.42	0.86
	Kaempferol-3-*O*-derivative (probably with a Hydroxycinnamic acids derivative in C3)	38.55	0.35
	Myrcetin	41.3	0.44
	Trycetin	43.18	2.33
	Luteolin	44.35	6.98
	Hydroxycinnamic acid derivative 1	50.84	2.00
	Hydroxycinnamic acid derivative 2	53.77	3.11
	Hydroxycinnamic acid derivative 3	54.44	9.35
BB Romania, April 2016	Herbacetin-3-*O*-glycoside	36.27	4.30
	Quercetin-3-*O*-glycoside	36.98	2.98
	Kaempferol-3-*O*-glycoside	38.38	2.85
	Herbacetin-3-*O*-glycoside	40.36	0.43
	Herbacetin-3-*O*-glycoside	42.17	0.27
	Hydroxycinnamic acid derivative 1	43.87	0.25
	Hydroxycinnamic acid derivative 2	45.17	1.31
	Hydroxycinnamic acid derivative 3	47.10	0.80
	Hydroxycinnamic acid derivative 4	47.21	1.18
	Hydroxycinnamic acid derivative 5	49.61	1.94
	Hydroxycinnamic acid derivative 6	50.75	1.74
	Hydroxycinnamic acid derivative 7	52.42	4.64
	Hydroxycinnamic acid derivative 8	53.68	0.43
BB Romania, June 2016	Kaempferol-3-*O*-glycoside	35.73	0.90
	Quercetin-3-*O*-glycoside	36.48	1.08
	Quercetin-3-*O*-glycoside	38.13	0.81
	Isoramnethin-3-*O*-glycoside	40.81	0.91
	Hydroxycinnamic acid derivative 1	43.48	0.61
	Hydroxycinnamic acid derivative 2	44.85	1.72
	Hydroxycinnamic acid derivative 3	45.62	0.28
	Hydroxycinnamic acid derivative 4	47.10	0.27
	Hydroxycinnamic acid derivative 5	47.57	0.24
	Hydroxycinnamic acid derivative 6	49.24	0.17
	Hydroxycinnamic acid derivative 7	50.52	0.26
	Hydroxycinnamic acid derivative 8	52.11	0.53
	Hydroxycinnamic acid derivative 9	53.16	0.33
	Hydroxycinnamic acid derivative 10	54.24	0.49
	Hydroxycinnamic acid derivative 11	55.10	0.44
